# A GoldenBraid-Compatible Virus-Based Vector System for Transient Expression of Heterologous Proteins in Plants

**DOI:** 10.3390/v14051099

**Published:** 2022-05-20

**Authors:** Helena Plchová, Tomáš Moravec, Noemi Čeřovská, Zuzana Pobořilová, Jakub Dušek, Kateřina Kratochvílová, Oldřich Navrátil, Jiban Kumar Kundu

**Affiliations:** 1 Laboratory of Virology, Centre for Plant Virus Research, Institute of Experimental Botany of the Czech Academy of Sciences, 16500 Prague, Czech Republic; plchova@ueb.cas.cz (H.P.); cerovska@ueb.cas.cz (N.Č.); poborilova@ueb.cas.cz (Z.P.); dusek@ueb.cas.cz (J.D.); kratochvilova@ueb.cas.cz (K.K.); navratil@ueb.cas.cz (O.N.); 2 Department of Plant Protection, Czech University of Life Sciences, 16500 Prague, Czech Republic; 3 Department of Experimental Plant Biology, Faculty of Science, Charles University in Prague, 12843 Prague, Czech Republic; 4 Plant Virus and Vector Interactions, Centre for Plant Virus Research, Crop Research Institute, 16106 Prague, Czech Republic

**Keywords:** Potato virus X, PVX vector, GoldenBraid, transient expression, *Nicotiana benthamiana*

## Abstract

We have developed a Potato virus X (PVX)-based vector system compatible with the GoldenBraid 2.0 (GB) cloning strategy to transiently express heterologous proteins or peptides in plants for biotechnological purposes. This vector system consists of three domestication vectors carrying three GB parts—the cauliflower mosaic virus (CaMV) 35S promoter with PVX upstream of the second subgenomic promoter of the PVX coat protein (PVX CP SGP), nopaline synthase (NOS) terminator with PVX downstream of the first PVX CP SGP and the gene of interest (GOI). The full-length PVX clone carrying the sequence encoding a green fluorescent protein (GFP) as GOI was incorporated into the binary GB vector in a one-step reaction of three GB parts using the four-nucleotide GB standard syntax. We investigated whether the obtained vector named GFP/pGBX enables systemic PVX infection and expression of GFP in *Nicotiana benthamiana* plants. We show that this GB-compatible vector system can be used for simple and efficient assembly of PVX-based expression constructs and that it meets the current need for interchange of standard biological parts used in different expression systems.

## 1. Introduction

Plant expression systems are cost-effective and highly productive platforms for protein expression, without endotoxins and with low risk of contamination [1,2,3]. Proteins can be expressed in plants and plant cells either by development of stably transformed lines or by transient expression systems. Plant viruses, especially viruses with RNA genomes, are suitable for high-level transient expression in plants because they replicate in high titres in infected cells, their relatively small genomes can be easily manipulated, and infection of plants with viruses is faster than obtaining stably transformed plants. In addition, plant viruses are considered potentially safe platforms for the production of vaccines and as delivery systems for therapeutic and contrast agents in animal and human therapy [4,5].

Potato virus X (PVX) a 6.4 kb plus-sense ssRNA genome, is the type member of the genus *Potexvirus* in the family *Alphaflexiviridae* [6]. PVX and other potexviruses have significant potential for protein expression in biotechnology. The vector based on the infectious full-length clone of PVX is widely used for transient expression of foreign proteins in plants. It can be used in two different ways—for the production of single full-length proteins (although, there are limitations on the size of sequences that can be expressed) [7,8] or for the presentation of epitopes on the surface of virus particles [9]. Fusions with PVX CP generally do not affect replication or assembly of the recombinant virus and therefore allow expression at high concentrations. Virus particle assembly is essential for long-distance movement, and the N-terminus plays a crucial role [10]. Previously, the foreign coding sequences were fused to the N-terminus of PVX CP [11,12,13], which is known to be exposed at the surface of the virion. However, in addition to the N-terminus of PVX CP, the C-terminus of PVX CP has also been used for fusion protein expression in plants. The C-terminal constructs were often unstable, the fusion proteins accumulated only in the inoculated leaves, and these recombinant viruses were not able to spread systemically [14,15,16,17].

Several strategies have been developed to design transient expression vectors derived from PVX [18]. These vectors are based on the use of a duplicated subgenomic promoter (SGP) [8], a single transcription unit encoding the gene CP, and the desired antigen separated by the foot-and-mouth disease virus (FMDV) catalytic peptide 2A [9], a bicistronic mRNA, containing an internal ribosome entry site [19], a substitution of the triple gene block (TGB) and CP genes with the sequence encoding a green fluorescent protein (GFP) [20], or a hybrid viral vector [21]. Because of genetic instability due to promoter duplication, PVX-based vectors have been stabilized by combining heterologous SGP-like sequences with an N-terminal PVX CP deletion [22]. Moreover, PVX-based vectors containing two additional SGPs can be used for the simultaneous expression of two foreign proteins [23]. Another shorter self-replicating PVX-based vector containing an expression cassette for the P24 silencing suppressor from grapevine leafroll-associated virus-2 was developed [24] and used for protein expression [25,26].

To enable rapid and easy construction of plant virus-based expression vectors, we have modified the infectious PVX clone according to the GoldenBraid (GB) standard syntax [27,28,29], simplifying its use for protein expression and leading to an exchange of ready-to-use and standardized genetic elements. Theoretically, any gene of interest (GOI) flanked by the correct barcodes can be easily linked to the PVX genome to generate a PVX-based vector that enables rapid transient expression of GOI. The full-length PVX clone carrying the GFP-encoding sequence was assembled from the three GB parts using the four-nucleotide GB standard syntax in the binary GB vector. We investigated whether the resulting PVX GB vector GFP/pGBX enables systemic PVX infection and expression of GFP in *Nicotiana benthamiana* plants. We have shown that this GB-compatible vector system can be used for simple and efficient assembly of PVX-based expression clones of target genes.

## 2. Materials and Methods

### 2.1. Viral and Plant Material, Vectors

The binary vector pGR106 carrying the full-length Potato virus X clone was kindly provided by D.C. Baulcombe (The Sainsbury Laboratory, Norwich, UK), and used to construct a GB-compatible PVX-based vector. *Nicotiana benthamiana* plants were grown for five weeks under controlled conditions (20–25 °C/15–20 °C day/night, 16 h/8 h-light/dark regime). The GB domestication (pUPD2) and destination (pDGB3α2) vectors were a kind gift of D. Orzaez (Plant Genomic and Biotechnology Lab, IBMCP, Valencia, Spain) [29]. The pUPD2b vector containing the two *BsmB*I cleavage sites with CTCG overhangs is a derivative of the vector pUPD2 (*BsmB*I cleavage sites CTCG and CTCA) prepared in our laboratory to allow backward compatibility with primers designed for the original pUPD.

### 2.2. Domestication of GB Parts 35S-PVX 0_17-2 and PVX-NOS 18_19 into the pUPD2b Vector

**Figure 1:**
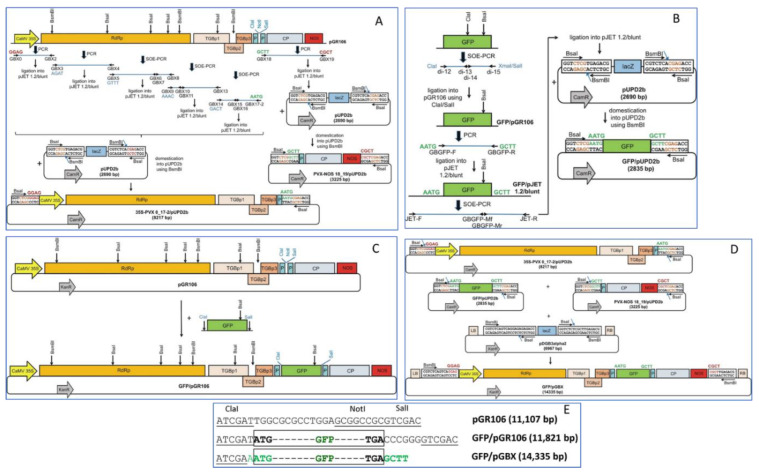
(**A**) Domestication of two parts of the PVX genome containing the CaMV 35S promoter (35S-PVX 0_17-2) and the NOS terminator (PVX-NOS 18_19) into the pUPD2b vector. The internal restriction sites *Bsa*I and *BsmB*I are removed in the original PVX sequence (the vector pGR106) by PCR and splicing by overlap extension PCR (SOE-PCR). The separated fragments of 35S-PVX 0_17-2 are linked to a 4-nucleotide barcode (shown in blue), ligated into the pJET 1.2/blunt vector, and domesticated into the pUPD2b vector using the *BsmB*I enzyme. Similarly, the PVX-NOS 18–19 fragment is domesticated into the pUPD2b vector by ligation into the pJET 1.2/blunt vector. The GB barcodes for GFP cloning and assembly into the destination vector pDGB3α2 are shown in green and red, respectively. The blue arrow indicates the cleavage site of *BsmB*I. CaMV 35S (Cauliflower mosaic virus 35S promoter), RdRp (RNA-dependent RNA-polymerase), TGBp1-3 (triple gene block proteins 1–3), P (PVX CP subgenomic promoter), CP (coat protein); NOS (nopaline synthase terminator); CamR (chloramphenicol resistance gene), lacZ (beta-galactosidase). (**B**) Construction of GFP/pGR106 expression vector and domestication of GFP into the pUPD2b vector. The gene encoding GFP (green fluorescent protein) is amplified by splicing by overlap extension PCR (SOE-PCR) and specific primers (di-12/di-15) to remove the internal *Cla*I site. The PCR product flanked by *Cla*I and *Sal*I sites (both in blue) is ligated into vector pGR106. The GB barcodes AATG and GCTT (both in green; for assembly with PVX GB parts in the destination vector pDGB3α2) are introduced using PCR and GB specific primers. After ligation of the PCR product into pJET 1.2/blunt, the internal *Bsa*I site in the GFP sequence is removed by SOE-PCR and GFP is ligated again into the pJET 1.2/blunt vector. GFP is then domesticated into the pUPD2b vector using the *BsmB*I enzyme. The blue arrow indicates the cleavage site of *BsmB*I. CamR, chloramphenicol resistance gene; lacZ, beta-galactosidase. (**C**) Schematic overview of the created GFP expressing vector—GFP/pGR106. PVX-based vector pGR106 with multiple cloning sites (MCS) containing sites for *Cla*I, *Not*I, and *Sal*I. These are used for a restriction enzyme-based insertion of a gene of interest, a green fluorescent protein (GFP), resulting in the vector GFP/pGR106. The internal restriction sites *Bsa*I and *BsmB*I are present in the vector. (**D**) Schematic overview of the created GFP-expressing vector—GFP/pGBX. The full-length PVX clone expressing GFP, adapted from the GB standard syntax. Three GB parts domesticated in pUPD2b, 35S-PVX 0_17-2 (barcodes GGAG/AATG), GFP (barcodes AATG/GCTT) and PVX-NOS 18_19 (barcodes GCTT/CGCT) are incorporated into the destination vector pDGB3α2 in a one-step reaction using *Bsa*I. The internal restriction sites *Bsa*I and *BsmB*I are removed from each fragment and MCS is absent. CaMV 35S, Cauliflower mosaic virus 35S promoter; RdRp, RNA-dependent RNA-polymerase; TGBp1-3, triple gene block proteins 1–3; P, PVX CP subgenomic promoter; MCS, multiple cloning site; CP, coat protein; NOS, nopaline synthase terminator; CamR, chloramphenicol resistance gene; KanR, kanamycin resistance gene; *Bsa*I and *BsmBI*, internal restriction sites; GFP, green fluorescent protein; lacZ, beta-galactosidase. (**E**) Comparison of insertion sites of expression vectors. The first row refers to a portion of the pGR106 vector with *Cla*I, *Not*I, and *Sal*I sites. The second row shows the insertion of GFP downstream of the *Cla*I site into the pGR106 vector. The third row shows the GFP sequence flanked by GB barcodes AATG and GCTT (in light green), which must be aligned according to the GB assembly. Due to AATG barcode, the *ClaI* site was interrupted and the *Not*I and *Sal*I sites were removed in the GFP/pGBX vector.

The individual fragments of the PVX construct (GBX0/2, GBX3/4, GBX5/6, GBX7/8, GBX9/10, GBX11/13, GBX14/15, GBX16/17-2, and GBX18/19) were amplified by PCR from the binary vector pGR106 (Figure 1) with the GB-adapted primers GBX (Supplementary Table S1). The GBX primers (with the exception of GBX0, GBX17-2, GBX18, and GBX19) were designed to contain single mismatches to disrupt the internal *BsmB*I or *Bsa*I sites (IIS type restriction enzymes used in GB cloning). Most GBX primers carry a *BsmB*I restriction site and four-nucleotides as sticky ends, that allow proper assembly of fragments into 35S-PVX 0_17-2 and PVX-NOS 18_19 parts. PCR reactions consisted of 1 μL pGR106 (20 ng), 10 μL 5× HF buffer, 1 μL 10 mM dNTPs, 1 μL of each forward and reverse primer (50 μM) (Supplementary Table S1), 0.25 μL Phusion High-Fidelity DNA Polymerase (2 U/μL) (Thermo Fischer Scientific, Waltham, MA, USA), and dH_2_O to the final volume of 50 μL. To obtain fragments by splicing by overlap extension PCR (SOE-PCR), two PCR products were purified using the High Pure PCR Product Purification Kit (Roche, Basel, Switzerland), mixed in an equimolar ratio, and added to the reaction mixture. The SOE-PCR reactions were performed in a final volume of 50 μL and contained 10 μL 5× HF buffer (in the case of GBX5/8 and GBX14/17-2 products) or 10 μL 5× GC buffer (in the case of GBX9/13 product), 1 μL 10 mM dNTPs, 0.5 μL Phusion High-fidelity DNA Polymerase (2 U/μL) (Thermo Fischer Scientific, Waltham, MA, USA), and 1 μL of the respective forward and reverse primers (50 μM). The SOE-PCR was performed with the following settings: denaturation phase at 98 °C for 30 s, followed by 35 cycles at 98 °C for 10 s, 60 °C for 30 s, 72 °C for 1 min (in the case of GBX5/8 product), 72 °C for 40 s (in the case of GBX9/13 product), or 72 °C for 30 s (in the case of GBX14/17-2 product), and final extension at 72 °C for 10 min. The first ten cycles were performed without the primers. The amplified products were purified using the High Pure PCR Product Purification Kit (Roche, Switzerland) and then cloned into the pJET 1.2/blunt vector (Thermo Fischer Scientific, Waltham, MA, USA), transformed into *Escherichia coli* (*E. coli*) DH5α using the heat-shock method [30], isolated and verified by sequencing. The individual fragments of the PVX construct (GBX0/2, GBX3/4, GBX5/8, GBX9/13, and GBX14/17-2) were assembled in a one-step reaction using *BsmB*I and T4 ligase in the pUPD2b vector to generate 35S-PVX 0_17-2/pUPD2b. The same restriction enzymes were used to clone the GBX18/19 fragment into the pUPD2b vector to obtain PVX-NOS 18_19/pUPD2b. Both vectors 35S-PVX 0_17-2/pUPD2b and PVX-NOS 18_19/pUPD2b were transformed into *E. coli* DH5α, isolated and verified by sequencing.

### 2.3. Construction of the GFP Expressing Vector GFP/pGR106

To clone GFP into pGR106, di-primers 12–15 (Supplementary Table S1) were designed and used to generate two fragments of GFP (Figure 1B,C). The di-primers contain single mismatches to disrupt the internal *Cla*I site and introduce *Cla*I and *Sal*I restriction sites for cloning. The PCR reaction consisted of 2 μL pGR2i-dCP-GFP (20 ng) (GenBank accession number KF981446), 10 μL 5× GC buffer, 1 μL 10 mM dNTPs, 1 μL of each forward and reverse primer (50 μM), 0.5 μL of Phusion High-fidelity DNA Polymerase (2U/μL), and dH_2_O to give a volume of 50 μL. PCR reaction conditions are given in Supplementary Table S1. The SOE-PCR was performed to obtain full-length GFP with the following settings: denaturation phase at 98 °C for 30 s, followed by ten cycles at 95 °C for 15 s, 62 °C for 30 s, 72 °C for 40 s, 25 cycles at 95 °C for 15 s, 60 °C for 30 s, 72 °C for 40 s, and final extension at 72 °C for 10 min. The first ten cycles were performed without primers. The amplified GFP with *Cla*I/*Sal*II restriction sites inserted was then cloned into vector pGR106, transformed into *E. coli* DH5α by the heat-shock method [30], isolated, and verified by sequencing.

### 2.4. Domestication of GFP into the pUPD2b Vector

The GFP sequence was amplified by PCR from the vector GFP/pGR106 with the GB-adapted primers GBGFP-F and GBGFP-R (Figure 1B) carrying the four-nucleotides AATG and GCTT, respectively. The PCR reaction consisted of 1 μL GFP/pGR106 (20 ng), 10 μL 5× HF buffer, 1 μL 10 mM dNTPs, 1 μL of each forward and reverse primer (50 μM), 0.5 μL Phusion High-fidelity DNA Polymerase (2 U/μL) and dH_2_O to a final volume of 50 μL. The PCR reaction conditions are listed in Supplementary Table S1. The amplified GFP was then cloned into vector pJET 1.2/blunt, transformed into *E. coli* DH5α by the heat-shock method [30], isolated and verified by sequencing. To disrupt the internal *Bsa*I site in the GFP sequence within the GFP/pJET 1.2/blunt vector, two GFP fragments were amplified with primers JET-F/GBGFP-Mr and GBGFP-Mf/JET-R (Supplementary Table S1) and HF-buffer. To obtain full-length GFP the SOE-PCR was performed with the following settings: denaturation phase at 98 °C for 15 s, followed by ten cycles at 98 °C for 15 s, 62 °C for 30 s, 72 °C for 30 s, 25 cycles at 98 °C for 15 s, 55 °C for 25 s, 72 °C for 30 s, and final extension at 72 °C for 10 min. The amplified product was cloned into vector pJET 1.2/blunt, transformed into *E. coli* DH5α using the heat-shock method [30], isolated and verified by sequencing. GB-compatible GFP with AATG and GCTT sticky ends was then introduced into the pUPD2b vector in a one-step reaction with *BsmB*I and T4 ligase. The resulting vector GFP/pUPD2b was transformed into *E. coli* DH5α, isolated and verified by sequencing.

### 2.5. Construction of the GFP Expressing GB-Vector GFP/pGBX

To construct the GB PVX-based vector GFP/pGBX expressing GFP, the GB parts 35S-PVX 0_17-2, PVX-NOS 18_19, and GFP, all cloned into the pUPD2b vector, were mixed together in the presence of the binary destination vector pDGB3α2, *Bsa*I and T4 ligase and incubated in cyclic restriction-ligation reaction. The GFP/pGBX expression vector (Figure 1D) was transformed into *E. coli* DH5α and checked for sequence accuracy after its isolation.

### 2.6. Transformation of Agrobacterium Tumefaciens and Plant Inoculation

GFP/pGBX and GFP/pGR106 vectors were transformed into *A. tumefaciens* GV3101 using the heat-shock method [30]. *A. tumefaciens* harbouring these vectors were selected on Luria-Bertani (LB)-agar plates supplemented with antibiotics (50 μg/mL kanamycin and 10 μg/mL rifampicin). The bacterial suspension for agroinfiltration of plants was prepared by growing the bacteria in the LB medium containing 50 μg/mL kanamycin and 10 μg/mL rifampicin at 28 °C for 2 days. Three 5-week-old *N. benthamiana* plants were inoculated with 1-mL injection syringe in each experiment [31], and all experiments were performed in triplicate.

### 2.7. Analysis of GB PVX Infection by RT-PCR

RT-PCR was performed as described by Čeřovská et al. 2003 [32] with some modifications. GFP/pGBX and GFP/pGR106 systemic leaves of *N. benthamiana* were harvested at 21 days post-inoculation (dpi). Leaves were homogenized 1: 10 (*w*:*v*) in a buffer (1.59 g Na_2_CO_3_, 2.93 g NaHCO_3_, 0.2 g NaN_3_, dH_2_O to a the volume of 1 L; pH 9.6) and briefly centrifuged. The tubes were coated with 50 µL of the plant extracts overnight at 4 °C, washed with phosphate-buffered saline containing 0.05% Tween 20 (4 × 100 µL PBST), and dried at 37 °C. The RT reaction was performed in the sap coated tubes using M-MLV reverse transcriptase (Promega, Madison, WI, USA) and di-4 primer binding near the 3′-end of the PVX CP gene. The region between TGBp2 and the CP gene was amplified using the primers GBX16 and di-10 (Supplementary Table S1) and Dream Taq Polymerase (Thermo Fisher Scientific, Waltham, MA, USA) under the following conditions: denaturation at 95 °C for 3 min followed by 40 cycles (denaturation at 95 °C for 30 s, primer annealing at 59 °C for 30 s and synthesis at 72 °C for 1 min 30 s) with the final extension at 72 °C for 10 min. The amplified products were analysed by 1% (*w*/*v*) agarose gel electrophoresis performed at a constant voltage of 100 V for 30 min (Bio-Rad, Hercules, CA, USA). The PCR product was then purified and sequenced using a commercial service (Eurofins Scientific, Val Fleuri, Luxembourg).

### 2.8. GFP Fluorescence Imaging in Plants

GFP fluorescence imaging was performed by illuminating the whole plants under UV LED light (400 nm). Fluorescence was observed and photographed through the yellow (Roscolux 12: straw for GFP) excitation filter using an Olympus digital camera (OLYMPUS PEN Lite E-PL3) and Zeiss Pancolar 50/1.8 lens. Images were taken at 4, 6, 11, 17, and 21 dpi.

### 2.9. Analysis of Infection of GB PVX by the Plate-Trapped Antigen Enzyme-Linked Immunosorbent Assay (PTA-ELISA)

One hundred µL of the leaf extracts from three individual plants (1: 10 (*w*:*v*) in coating buffer) were added to the wells of the 96-well microtiter plate—each sample in duplicate—and the plates were incubated overnight at 4 °C. After incubation, the plates were washed with PBST (3 × 200 µL). For detection of PVX CP, 100 µL of a primary rabbit polyclonal anti-PVX CP antibody (dilution 5 µg/mL in conjugation buffer containing PBST, 2% polyvinylpyrrolidone, 0.2% ovalbumin, pH 7.4) was added and plates were incubated for 2 h at 37 °C. Plates were washed again three times with 200 µL PBST and 100 µL alkaline phosphatase conjugated secondary anti-rabbit IgG (Sigma-Aldrich, St Louis, MI, USA, dilution 1:10,000 in conjugation buffer) was added and plates were incubated again overnight at 37 °C. After another washing step, 100 µL of p-nitrophenyl-phosphate (pNPP; 1 mg/mL substrate buffer containing 1 M diethanolamine, pH 9.8) was added and the plates were incubated at 37 °C for 1 h. Absorbance was measured at 405 nm using an Infinite F200 PRO plate reader (Tecan, Männedorf, Switzerland). Excel software was used to analyze the data.

### 2.10. Purification of GB PVX Particles

To ensure that virus particles were produced in plants, systemically infected leaves of *N. benthamiana* expressing GFP were harvested on day 19 after agroinfiltration and virus particles were isolated. Twenty grams of systemically infected leaves were homogenized in 40 mL of 0.05 M sodium-potassium phosphate buffer (9 g Na_2_HPO_4_, 1.7 g KH_2_PO_4_ per 750 mL of distilled water, pH 7.3) supplemented with 0.01 M EDTA, 0.5 % (*v*/*v*) DIECA, 0.5% (*w*/*v*) Na_2_SO_3_, 0.02% (*w*/*v*) NaN_3_, 1:100 (*v*/*v*) PMSF, and one drop of octanol. The extract was passed through a cheesecloth and centrifuged at 10,000 rpm at 4 °C for 10 min. The supernatant was collected and 40 mL was mixed with 0.8 mL of Triton X-100 (final concentration of 2%) and stirred on ice for 20 min. Then, 2.4 g PEG 6000 and 0.8 g NaCl were added to the supernatant, stirred again on ice for 1 h, and centrifuged at 10,000 rpm at 4 °C for 10 min. The sediment was dissolved in 10 mL of 0.05 M sodium-potassium-phosphate buffer containing one drop of PMSF, stirred on ice for 1 h, and centrifuged at 10,000 rpm at 4 °C for 10 min. The suspension was stored overnight at 4 °C and further purified by ultracentrifugation on a sucrose cushion pad consisting of 5 mL 30% sucrose in 0.05 M sodium-potassium-phosphate buffer at 27,000 rpm at 4 °C for 2 h. The sediment was dissolved in 0.5 mL of 0.05 M sodium-potassium-phosphate buffer containing one drop of PMSF and centrifuged at 10,000 rpm at 4 °C for 10 min. The concentration of PVX particles was determined using the Lambert-Beer law and the extinction coefficient of 2.97 cm^−1^ mg^−1^ mL (at 260 nm). The purified virus particles were then visualized by negative staining with 5% uranyl acetate under a transmission electron microscope FEI Morgagni 268D (Field Electron and Ion Company, Hillsboro, OR, USA).

## 3. Results

### 3.1. Construction of GFP Expressing GB-Compatible PVX-Based Vector GFP/pGBX

The PVX-based vector system was developed to facilitate cloning and rapid construction of expression constructs for transient expression in plants. The PVX-based vector system is compatible with the versatile GoldenBraid (GB) standard syntax. Analysis of the complete PVX sequence in the vector pGR106, including CaMV 35S promoter and NOS terminator, revealed the presence of “forbidden“ restriction sites, namely three *BsmB*I and four *Bsa*I sites (Figure 1A). Three *Bsa*I and two *BsmB*I sites were located in the RdRp gene, whereas one BsaI and one *BsmB*I site were found in the coding sequences of TGBp1 and TGBp2, respectively. To allow domestication of the PVX sequence of pGR106 in pUPD2b, these internal restriction sites were removed by introducing point mutations (C369T, C1956T, G3201A, G3654A, C3987T in RdRp, C414T in TGBp1, and C171T in TGBp2) without altering the amino acid sequences of the encoded proteins. In addition, the four-nucleotide sticky ends used to connect each PCR fragment were designed to achieve “scarless“ assembly of 35S-PVX 0_17-2. Consistent with GB2.0 grammar and to allow cloning of the gene of interest, the resulting GB parts 35S-PVX 0_17-2 and PVX-NOS 18_19 domesticated in pUPD2b were flanked by four-nucleotide “barcodes” GGAG/AATG and GCTT/CGCT, respectively (Figure 1A). 

We also produced the unmodified PVX-based vector expressing GFP (GFP/pGR106) as a positive control (Figure 1B,C). The GFP gene was chosen as a reporter because it allows easy study of protein expression, PVX infection, and its spread. First, the internal restriction site *Cla*I was removed from the GFP sequence by using SOE-PCR and GFP-specific primers carrying the mutation C384A at the *Cla*I site (Figure 1B, Supplementary Table S1). The amplified GFP was then cloned into the pGR106 vector using the *Cla*I and *Sal*I sites. The gene of interest (GB part GFP) was flanked by AATG/GCTT “barcodes” using GB specific primers (Supplementary Table S1), amplified by PCR, and cloned into the pJET 1.2/blunt vector. Because the presence of an internal *Bsa*I site in the GFP sequence (Figure 1B) would interfere with direct cloning of the reporter gene into the GB destination vector pDGB3α2 using the *Bsa*I restriction enzyme, this internal restriction site was disrupted by introducing the silent mutation C648T using SOE-PCR. The product of SOE-PCR was again cloned into the pJET 1.2/blunt vector and then domesticated into pUPD2b. Finally, the GB parts 35S-PVX 0_17-2, PVX-NOS 18_19, and GFP domesticated in the pUPD2b vector were assembled into the GB destination vector pDGB3α2, resulting in the GB-compatible PVX-based vector GFP/pGBX (Figure 1D). A comparison of the insertion sites of the expression vectors is shown in Figure 1E. The GFP reporter was placed downstream of the *Cla*I site in both expression vectors—GFP/pGR106 and GFP/pGBX. However, in the latter, the *Cla*I site is interrupted due to the presence of the AATG “barcode”. In addition, the *Not*I and *Sal*I sites were removed.

### 3.2. Analysis of PVX Infection in N. benthamiana Plants

**Figure 2:**
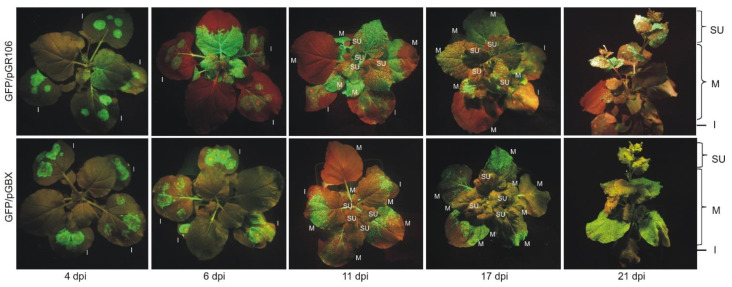
GFP expression in *N. benthamiana* plants visualized under UV light. Systemic infection of GFP/pGBX plants is delayed by 2 days (at 6 dpi) compared to GFP/pGR106 plants (4 dpi). Over time, the GFP/pGBX vector accumulated GFP mainly in the middle part of the plant, whereas GFP expression in GFP/pGR106 plants progressed throughout the plant to the SU leaves. Photographs were taken at 4, 6, 11, 17, and 21 dpi, respectively. Representative photos for GFP/pGR106 and GFP/pGBX plants were selected. The data shown on the right (I, M, SU) represent the distribution of the evaluated plant parts; dpi, days post-inoculation; I, inoculated leaves; M, systemic middle leaves; SU, systemic four upper leaves.

**Figure 3:**
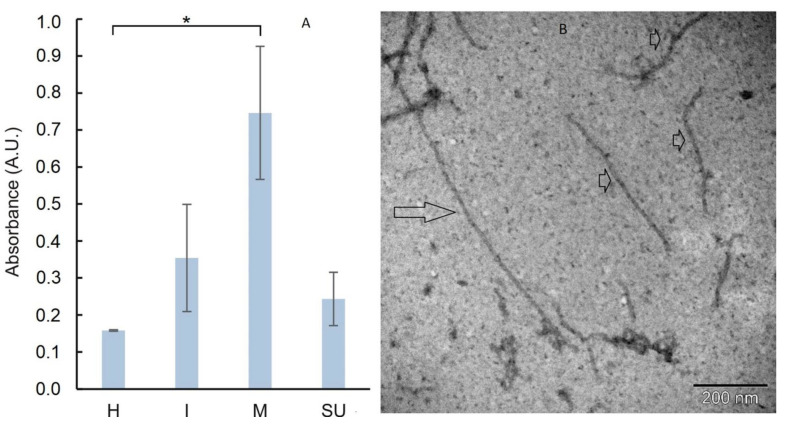
(**A**) PTA-ELISA analysis of PVX CP expression in GFP/pGBX inoculated plants at 21 dpi. The PVX CP was mainly present in the middle part of the plant. Means and standard deviations are presented. Asterisk indicates statistical significance. *, *p* < 0.05 (*t*-test: paired two-sample for means). H, healthy leaves; I, inoculated leaves; M, systemic middle leaves; SU, systemic four upper leaves. (**B**) Electron microscopy of PVX particles (small arrow—single particle around 515 nm × 13 nm and larger arrow—the aggregated particles), isolated from systemic GFP/pGBX leaves of *N. benthamiana*. This confirms that the deleted version of PVX CPs also forms viral particles. Bar = 200 nm.

**Figure 4:**
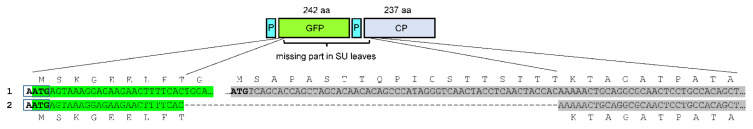
Alignment of the GFP/pGBX construct (1) and the RT-PCR GFP/pGBX product amplified from SU leaves at 21 dpi (2). The missing part (amino acids 9-242 of the GFP gene, PVX CP SGP, and the first 18 amino acids of PVX CP) is shown. The result of this deletion is the in-frame fusion of the first eight amino acids of GFP with the remainder of PVX CP. The ATG start codons of GFP and PVX CP are shown in bold, the GB barcode for the cloning of GFP is shown in frames. P, PVX CP subgenomic promoter.

We infected *N. benthamiana* plants with *A. tumefaciens* carrying either the GB-compatible construct GFP/pGBX or the control vector GFP/pGR106. First, we analyzed the plants by monitoring GFP expression under UV-light. The appearance of GFP-fluorescent zones on the inoculated leaves was evident for both vectors at 4 dpi (Figure 2). In the case of the GFP/pGR106 control, green fluorescence also appeared on the systemic leaves, whereas the GFP/pGBX vector showed the first signs of GFP fluorescence on the systemic leaves 2 days later, i.e., at 6 dpi. GFP expression at 11 dpi was more robust for the GFP/pGR106 vector; systemic 4 upper leaves (SU) showed green fluorescence in 100% of GFP/pGR106 plants (*n* = 9), whereas only 44% were detected in GFP/pGBX-infected plants (*n* = 4). Expression of GFP in inoculated leaves was weaker at 11 dpi than at 6 dpi in both vectors (Figure 2). Later in the infection (both 17 and 21 dpi), GFP expression in GFP/pGBX SU leaves was detected in 33% of plants (*n* = 3), while GFP/pGR106 SU leaves still showed 100% expression (*n* = 9). At both time points, almost no GFP fluorescence was seen on leaves inoculated with GFP/pGR106 or GFP/pGBX. GFP expression with the GFP/pGR106 vector spread throughout the plant to the SU leaves, whereas the GFP/pGBX vector accumulated GFP mainly in the middle part of the plant. Because PVX CP is required for both cell-to-cell and long-distance movement of the virus, expression of PVX CP was analyzed throughout the GFP/pGBX plant at 21 dpi. Inoculated leaves, systemic leaves from the middle part of the plants, and SU leaves were harvested and PTA-ELISA was performed (Figure 3A). The analysis confirmed the presence of PVX CP in all GFP/pGBX leaves analyzed, mainly in the middle part of the plants. This result is consistent with GFP expression detected under UV light (Figure 2). In addition, we confirmed that PVX-CPs also formed virus particles, as PVX particles were observed after isolation from systemic GFP/pGBX leaves (Figure 3B). To further analyze GFP expression in SU GFP/pGBX leaves, RT-PCR was performed with primers (Supplementary Table S1), amplifying the region between TGBp2 and the CP gene. At 21 dpi, control SU GFP/pGR106 leaves still yielded the expected full-length product of 1.2 kbp with the full-length GFP sequence as determined by DNA sequencing, SU GFP/pGBX leaves showed only a truncated version of the viral RNA. Sequencing of the RT-PCR GFP/pGBX product revealed a deletion of most of the GFP gene (amino acids 9–242), PVX CP SGP and the first 18 amino acids of PVX CP. The result of this deletion was an in-frame fusion of the first eight amino acids of GFP with the remainder of PVX CP (Figure 4). Therefore, the weaker expression of PVX CP, detected by PTA-ELISA in the SU leaves (Figure 3A), may be due to the difficulty in detecting the chimeric version of CP. Since PVX CP, which lacks the first 29 amino acids, can still assemble into intact particles [23], it is expected that isolated PVX particles also consist of the fusion mutant PVX CP.

### 3.3. Use of PVX as Inducer of Virus Inducing Gene Silencing (VIGS)

**Figure 5:**
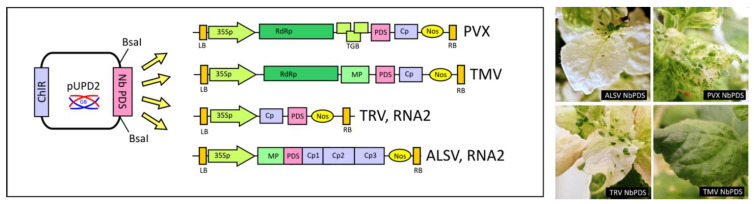
Schematic representation of the VIGS vector. The 206 bp long NbPDS gene fragment was first cloned into the universal domestication vector pUPD2.The PDS fragment was then used in four different GB assembly reactions to generate four infectious cDNA clones of the PVX, TMV, TRV, and ALSV viruses. LB, RB—left and right T-DNA edges; 35Sp—CaMV 35S promoter; RdRp—RNA-dependent RNA polymerase; TGB—triple gene block; PDS—fragment of Nb phytoene desaturase; CP—coat protein; Nos—*A. tumefaciens* nopaline synthase terminator; MP—movement protein. The diagram is not drawn to scale. Leaves of *Nicotiana benthamiana* showing the phenotype of photobleaching and symptoms of viral infection at 20 dpi.

An important advantage of the GoldenBraid platform is the use of standard syntax for parts that are interchangeable and reusable. Some RNA plant viruses, including PVX, are also known to be potent virus induced gene silencing (VIGS) triggers. We wanted to demonstrate the potential of the GoldenBraid-modified PVX vector not only for overexpression of proteins of interest, but also for use as a VIGS vector. In this experiment, we decided to silence the visible marker gene phytoene desaturase (NbPDS) in *Nicotiana benthamiana* by cloning a single NbPDS in parallel into different plant virus vectors, namely PVX, Tobacco mosaic virus-TMV [33], Tobacco rattle virus –TRV [34], and Apple latent spherical virus-ALSV [35]. Symptoms of virus infection and VIGS-induced photobleaching were observed on the upper leaves 20 days after infiltration (Figure 5). Both ALSV and TRV showed the strongest photobleaching phenotype, PVX showed intermediate photobleaching, while plants infected with TMV showed no photobleaching phenotype. The upper leaves of PVX and TRV infected plants were deformed, which corresponds to the symptoms of virus infection. The stability of the NbPDS insert in all four viruses was verified by RT-PCR from upper symptomatic leaves. This experiment shows that the modified PVX vector can also be used for VIGS of genes in *N. benthamiana* and probably in other plants susceptible to PVX infection. It also demonstrates the advantage of the GoldenBraid/MoClo, where the same NbPds2 fragment can be cloned into multiple viral vectors without having to adapt cloning strategies or design primers specifically for each virus. 

## 4. Discussion

Plant virus-based vectors are often used as an alternative to stable genetic transformation of plants for the transient expression of valuable heterologous proteins or peptides. Advances in synthetic biology have improved methods for constructing the viral infectious clone, which is the main component of the viral vector. In addition to restriction endonuclease-based methods such as Golden Gate [36,37] or GoldenBraid [27,29], overlap-based methods such as Gibson [38] or In-Fusion [39], which eliminate the need for restriction enzymes, can also be used for assembly. However, these two methods are neither strictly modular nor suitable for synthetic biology of plants. Traditional cloning of sequences encoding for heterologous proteins into the viral vector depends on the restriction sites available in the vector, which may vary depending on the expression system used. The GB system based on Golden Gate, allows cloning of standardized base elements that follow standard assembly rules. In our previous work, we have developed a series of novel plasmids that are compatible with the GB standard syntax and demonstrate the ability of GB to rapidly assemble more complex genetic constructs [40]. In addition, we have demonstrated the use and versatility of GB-produced vectors for the transient expression of fluorescent proteins in tobacco BY-2 cells [41]. In the present study, we assembled and adapted the infectious clone of PVX according to GB standard syntax to facilitate its use for protein expression. Another advantage of the GoldenBraid conversion is that the infectious clone can be inserted into any GoldenBraid-compatible plasmid backbone. The original clone was based on the pGREEN backbone and was thus restricted to Agrobacterium GV3103 with the pSoup helper plasmid. The backbones of the existing GB plasmids also include pCAMBIA vectors and pLX vectors with the broad replication origin pBBR1 with low copy number in *E.coli.* It thus offers flexibility in the choice of plasmid copy number, antibiotic selection marker and Agrobacterium strain. It also allows the addition of multiple gene cassettes with additional functions on the same plasmid with the infectious clone.

To investigate the possibility of expressing a foreign gene with the GB-compatible PVX vector in *N. benthamiana* plants, we chose GFP as the reporter gene. We removed the unwanted internal restriction sites *BsmB*I and *Bsa*I from the PVX genome by site-directed mutagenesis and then incorporated two separate parts of the PVX genome (35S-PVX 0_17-2 and PVX-NOS 18_19) into the pUPD2b domestication vector to introduce four-nucleotide GB barcodes for subsequent GFP cloning. The GFP was equipped with the same barcodes and also cloned into pUPD2b. The full-length PVX clone carrying the sequence coding for GFP was assembled into the binary GB vector pDGB3α2 using four-nucleotide GB standard syntax and named GFP/pGBX. The GFP gene was placed downstream of the *Cla*I site, which was simultaneously interrupted, to ensure the same distance from the duplicated PVX CP SGP as in the case of GFP in pGR106. Both vectors clearly expressed GFP at the same time in inoculated leaves. However, the GFP/pGBX vector showed the first signs of GFP expression on systemic leaves two days later than the GFP/pGR106 vector. Mutations in RdRp, TGBp1, or TGBp2 could not be the reason for this delay, as the mutations introduced into the PVX genome to remove the internal restriction sites did not change the amino acid sequences of the encoded proteins. The TGB proteins and CP are required for cell-to-cell and long-distance movement [42]. Many (+)ssRNA viruses use subgenomic RNAs (sgRNAs) for the expression of their 3′-terminal genes, as the only RdRp can be translated directly from genomic RNA [43,44]. PVX TGBp1-3 and CP are translated by sgRNAs. Two major sgRNAs have their 5′-termini upstream of the TGB and CP genes, and the third minor sgRNA has its 5′-terminus upstream of the TGBp2 gene [45,46]. Alignment of the potexvirus SGP sequences revealed a conserved octanucleotide element upstream of the transcription initiation site, which is important for sgRNA accumulation [45,47,48]. None of the mutations we introduced into the PVX genome altered this element, so accumulation of sgRNAs should not be affected. However, a previous study has shown that a vector carrying GFP under the duplicated PVX SGP and the N-terminal PVX CP, which lacks the first 29 amino acids, is slower to produce systemic infection due to insufficient yield of the CP sgRNA, which cannot provide enough CP for normal infection. The yield of CP sgRNA under the heterologous Bamboo mosaic virus (BaMV) SGP was higher and thus the infection rate was comparable to that of the original vector, suggesting that additional sequences to the SGP contribute to efficient sgRNA production [22]. The slower systemic infection of GFP/pGBX compared to the original GFP/pGR106 could probably be explained by changes in the sequence between the duplicated PVX SGPs (cloning sites were removed and GB barcodes flanking GFP were introduced) or by the vector backbone, being about 2.5 kbp longer than in the case of GFP/pGR106.

The PVX genome can accommodate large heterologous sequence insertions, such as the 1.8 kbp GUS gene [49] or the 1.8 kbp human GAD65 gene [7]. Usually, the expression of the heterologous gene is controlled by an additional copy of PVX SGP so that a fourth sgRNA, consisting of the heterologous gene and CP, is produced during infection. It is already known that in vectors containing duplicated SGPs, the inserted gene tends to be deleted due to template switching at sequence repeats by viral replicase [7,49,50,51]. Deletion of the insert can occur during the initial infection and is more likely when the insert is large [7]. The stability of the viral vectors could be further improved either by insertion of heterologous SGP [52,53,54] or by combining the heterologous SGP with the N-terminal CP deletion mutant, as previously described for the PVX vector [22]. While GFP/pGR106 plants still retained the complete GFP sequence three weeks after inoculation and GFP expression progressed throughout the plant to the SU leaves, GFP/pGBX plants accumulated GFP mainly in the middle part of the plant and systemic GFP/pGBX leaves contained a deletion lacking the major part of the GFP gene, CP SGP, and 18 amino acids of CP. It appears that the construct adapted to GB PVX is more susceptible to loss of insertion during infection compared to GFP/pGR106. Therefore, further studies are needed to improve the stability of the vector system in plants and to increase its efficacy. 

## 5. Conclusions

In this work, we have shown that the constructed GoldenBraid-compatible PVX-based vector system can be used for the simple and efficient assembly of PVX-based expression constructs and that the assembled GB PVX vector enables systemic PVX infection and GFP expression despite the mutations and barcodes introduced into the PVX genome. However, the stability of the assembled expression should be considered and special care should be taken to apply foreign gene expression in the described vector system.

## Data Availability

All data included in the main text.

## Supplementary Materials

The following supporting information can be downloaded at: https://www.mdpi.com/article/10.3390/v14051099/s1, Table S1: List of primers used in this study.
