# Supplementary material for: A GoldenBraid-Compatible Virus-Based Vector System for Transient Expression of Heterologous Proteins in Plants
Source: Viruses. 2022 May 20;14(5):1099. doi: 10.3390/v14051099 (PMC9146717; doi:10.3390/v14051099)
Supplement: Supplementary file 1 [file viruses-14-01099-s001.zip › viruses-1675926-supplementary.pdf]

Communication

A Golden Braid-compatible virus-based vector system for transient expression of heterologous proteins in plant

Helena Plchová[1], Tomáš Moravec[1,*], Noemi Čeřovská[1], Zuzana Pobořilová[1], Jakub Dušek[1,2], Kateřina Kratochvílová[1,3], Oldřich Navrátil[1], Jiban Kumar Kundu[1,4*]

1- Laboratory of Virology - Centre for Plant Virus Research, Institute of Experimental Botany of the Czech Academy of Sciences, Rozvojová 313, 165 00 Prague 6, Czech Republic
2- Department of Plant Protection, Czech University of Life Sciences, Kamýcká 129, 165 00 Prague, Czech Republic
3- Department of Experimental Plant Biology, Faculty of Science, Charles University in Prague, Albertov 6, 12843 Prague, Czech Republic
4- Plant Virus and Vector Interactions - Centre for Plant Virus Research, Crop Research Institute, Drnovská 507, Prague 6, 161 06 Czech Republic.

Table S1. List of primer used in this study

Set	Primer	Sequence (5´-3´)[a]	PCR conditions
1	GBX0	gcgc**cgtctc**gctcgggagctcgcatgcctgcaggtca	denaturation 98°C 30s, 30 cycles (denaturation 98°C 10s, primer annealing 60°C 30s, synthesis 72°C 35s), extension 72°C 10min
	GBX2	gcgc**cgtctc***catctc*ttggttcaatggcaacat	
2	GBX3	gcgc**cgtctc***gagatg*tagccaggtaccccaaggaa	denaturation 98°C 30s, 30 cycles (denaturation 98°C 10s, primer annealing 59°C 30s, synthesis 72°C 1 min), extension 72°C 10min
	GBX4	gcgc**cgtctc***gaaacc*atttctgtgatgggcatgat	
3	GBX5	gcgc**cgtctc***ggtttc*cgggctggaaaaagagga	denaturation 98°C 30s, 28 cycles (denaturation 98°C 10s, primer annealing 60°C 30s, synthesis 72°C 1 min), extension 72°C 10min
	GBX6	cagaggag*ggtttc*atcttttgctt	
4	GBX7	caaaagat*gaaacc*ctcctctgggc	denaturation 98°C 30s, 25 cycles (denaturation 98°C 10s, primer annealing 60°C 25s, synthesis 72°C 20s), extension 72°C 10min
	GBX8	gcgc**cgtctc***ggtttc*acagtttatgaagacttcttttg	
	GBX9	gcgc**cgtctc***gaaacc*acgccagatgacatgtctg	

5	GBX10	gtgcggagt*catctc*ctgcataa	denaturation 98°C 30s, 25 cycles (denaturation 98°C 10s, primer annealing 60°C 25s, synthesis 72°C 20s), extension 72°C 10min
6	GBX11	ttatgcag*gagatg*actccgcac	denaturation 98°C 30s, 30 cycles (denaturation 98°C 10s, primer annealing 60°C 30s, synthesis 72°C 35s), extension 72°C 10min
	GBX13	gcgc**cgtctc**t*agtctc*gaaatcgaagccacagccag	
7	GBX14	gcgc**cgtctc**_agact_aactcaccggaagaagggca	denaturation 98°C 30s, 30 cycles (denaturation 98°C 10s, primer annealing 60°C 25s, synthesis 72°C 20s), extension 72°C 10min
	GBX15	tttggtgc*catctc*tgtaagctcc	
8	GBX16	agcttaca*gagatg*gcaccaaagc	denaturation 98°C 30s, 30 cycles (denaturation 98°C 10s, primer annealing 60°C 25s, synthesis 72°C 20s), extension 72°C 10min
	GBX17-2	gcgc**cgtctc**gctcgcatttcgatgctagctggtgctga	
9	GBX18	gcgc**cgtctc**gctcggcttcgccgatgaacggttaagttt	denaturation 98°C 30s, 30 cycles (denaturation 98°C 10s, primer annealing 60°C 30s, synthesis 72°C 40s), extension 72°C 10min
	GBX19	gcgc**cgtctc**gctcgagcgcccgatctagtaacatagatga	
10	di-12	cattatcgatatgagtaaaggagaagaactt	denaturation 98°C 30s, 35 cycles (denaturation 98°C 10s, primer annealing 57°C 30s, synthesis 72°C 25s), extension 72°C 10min
	di-13	cctccttgaaatctattccttttaac	
11	di-14	gttaaaaggaatagatttcaaggagg	denaturation 98°C 30s, 35 cycles (denaturation 98°C 10s, primer annealing 56°C 25s, synthesis 72°C 25s), extension 72°C 10min
	di-15	cattgtcgaccccgggtcaattatttacggccgcg	
12	GBGFP-F	gcgc**cgtctc**gctcgaatgagtaaaggagaagaacttttt	denaturation 98°C 30s, 30 cycles (denaturation 98°C 15s, primer annealing 60°C 25s, synthesis 72°C 25s), extension 72°C 10min
	GBGFP-R	gcgc**cgtctc**gctcgaagctcaattatttacggccgcgac	
13	JET-F	cgactcactatagggagagcggc	denaturation 98°C 30s, 30 cycles (denaturation 98°C 15s, primer annealing 55°C 25s, synthesis 72°C 25s, extension 72°C 10min
	GBGFP-Mr	ggaccatgt*gatctc*tcttttc	
14	GBGFP-Mf	gaaaaga*gagatc*acatggtcc	denaturation 98°C 30s, 30 cycles (denaturation 98°C 15s, primer annealing 55°C 25s, synthesis 72°C 25s, extension 72°C 10min
	JET-R	aagaacatcgattttccatggcag	
15	di-4	cgacctcgagtgacagctg	RT

16	di-10	ccgggatagtcaggcctgaagctgtggca	RT with GBX16: denaturation 95°C 3 min, 40 cycles (denaturation 95°C 30s, primer annealing 59°C 30s, synthesis 72°C 1 min 30s), extension 72°C 10min
17	Gb-NbPDS-F	gcgccgtctcgctcgaatgcccgcacttaacttcataaac	denaturation 98°C 30s, 30 cycles (denaturation 98°C 15s, primer annealing 60°C 25s, synthesis 72°C 25s), extension 72°C 10min
	Gb-NbPDS-Rv2	gcgccgtctcgctcacgaaccctcgatcttttttattc	

[a]*Restriction site BsmBI in bold; mutated restriction sites in upper-case and italics; restriction sites used for cloning in upper-case; four-nucleotide sticky ends underlined*
